# Quantitative ^18^F-AV1451 Brain Tau PET Imaging in Cognitively Normal Older Adults, Mild Cognitive Impairment, and Alzheimer's Disease Patients

**DOI:** 10.3389/fneur.2019.00486

**Published:** 2019-05-15

**Authors:** Qian Zhao, Min Liu, Lingxia Ha, Yun Zhou, Alzheimer's Disease Neuroimaging Initiative, Michael W. Weiner

**Affiliations:** Author Affiliations: UC San Francisco; University of Southern California; UC San Francisco University of Southern California Mayo Clinic, Rochester Mayo Clinic, Rochester; UC Berkeley; U Pennsylvania; USC; UC Davis; Brigham and Women's Hospital/Harvard Medical School Indiana University Washington University St. Louis University of Pennsylvania; Prevent Alzheimer's Disease 2020 (Chair) Siemens; Alzheimer's Association University of Pittsburgh Washington University St. Louis Cornell University; Albert Einstein College of Medicine of Yeshiva University; AD Drug Discovery Foundation; Acumen Pharmaceuticals; Washington University St. Louis; Northwestern University; National Institute of Mental Health; Brown University; Eli Lilly (Chair); BWH/HMS (Chair); University of Washington (Chair); Mayo Clinic, Rochester (Core PI) University of Southern California; UC San Diego; UC San Diego; UC San Diego; UC San Diego; UC San Diego; UC San Diego; UC San Diego; UC San Diego; UC San Diego; UC Davis (Core PI); UC Davis; UC San Diego; Mayo Clinic, Rochester (Core PI); Mayo Clinic, Rochester; University of London; UCLA School of Medicine; UCSF MRI; UC Davis; Mayo Clinic; Mayo Clinic; Mayo Clinic; Mayo Clinic; Mayo Clinic; Mayo Clinic; Mayo Clinic; UC Berkeley (Core PI); University of Michigan; University of Utah; Banner Alzheimer's Institute; Banner Alzheimer's Institute; University of Pittsburgh; UC Berkeley; Washington University St. Louis; Washington University St. Louis; Washington University St. Louis; Washington University St. Louis; UPenn School of Medicine; UPenn School of Medicine; UPenn School of Medicine; UPenn School of Medicine; UPenn School of Medicine; USC (Core PI); USC; USC; Indiana University; Indiana University; UC Irvine; Indiana University; Indiana University; Indiana University; Indiana University; UC San Francisco; UC San Diego; Prevent Alzheimer's Disease 2020; UC San Diego; National Institute on Aging; UC San Francisco; Brown University; National Institute of Mental Health; Cornell University; Johns Hopkins University; Richard Frank Consulting; Prevent Alzheimer's Disease 2020; National Institute on Aging; Oregon Health & Science University; University of Southern California; University of California - San Diego; University of Michigan; Mayo Clinic, Rochester; Baylor College of Medicine; Columbia University Medical Center; Washington University, St. Louis; University of Alabama - Birmingham; Mount Sinai School of Medicine; Rush University Medical Center; Wien Center; Johns Hopkins University; New York University; Duke University Medical Center; University of Pennsylvania; University of Kentucky; University of Pittsburgh; University of Rochester Medical Center; University of California, Irvine; University of Texas Southwestern Medical School; Emory University; University of Kansas, Medical Center; University of California, Los Angeles; Mayo Clinic, Jacksonville; Indiana University; Yale University School of Medicine; McGill Univ., Montreal-Jewish General Hospital; Sunnybrook Health Sciences, Ontario; U.B.C. Clinic for AD & Related Disorders; Cognitive Neurology - St. Joseph's, Ontario; Cleveland Clinic Lou Ruvo Center for Brain Health; Northwestern University; Premiere Research Inst (Palm Beach Neurology); Georgetown University Medical Center; Brigham and Women's Hospital; Stanford University; Banner Sun Health Research Institute; Boston University; Howard University; Case Western Reserve University; University of California, Davis - Sacramento; Neurological Care of CNY; Parkwood Hospital; University of Wisconsin; University of California, Irvine - BIC; Banner Alzheimer's Institute; Dent Neurologic Institute; Ohio State University; Albany Medical College; Hartford Hospital, Olin Neuropsychiatry Research Center; Dartmouth-Hitchcock Medical Center; Wake Forest University Health Sciences; Rhode Island Hospital; Butler Hospital; UC San Francisco; Medical University South Carolina; St. Joseph's Health Care; Nathan Kline Institute; University of Iowa College of Medicine; Cornell University; University of South Florida: USF Health Byrd Alzheimer's Institute; University of California, San Francisco; University of Southern California; UC San Francisco; University of Southern California; Mayo Clinic, Rochester; Brigham and Women's Hospital/ Harvard Medical School; UC Davis; Mayo Clinic, Rochester; UC Berkeley; Washington University St. Louis; Indiana University; Perelman School of Medicine, UPenn; USC; Perelman School of Medicine, University of Pennsylvania; UC San Francisco; Rehabilitation Institute of Chicago, Feinberg School of Medicine, Northwestern University; BWH/HMS (Chair); University of Washington (Chair); Core PI; Mayo Clinic, Rochester (Core PI); University of Southern California; UC San Diego; UC San Diego; UC San Diego; UC San Diego; UC San Diego; UC San Diego; UC San Diego; UC San Francisco; UC San Francisco; UC San Francisco; UC Davis (Core PI); UC San Diego; Mayo Clinic, Rochester (Core PI); Mayo Clinic, Rochester; Mayo Clinic; Mayo Clinic; Mayo Clinic; Mayo Clinic; Mayo Clinic; UC Berkeley (Core PI); University of Michigan; University of Utah; Banner Alzheimer's Institute; Banner Alzheimer's Institute; UC Berkeley; Washington University St. Louis; Washington University St. Louis; Washington University St. Louis; Perelman School of Medicine, UPenn; Perelman School of Medicine, UPenn; Perelman School of Medicine, UPenn; Perelman School of Medicine, UPenn; Perelman School of Medicine, UPenn; USC (Core PI); USC; USC; Indiana University; Indiana University; UC Irvine; Indiana University; Indiana University; Indiana University; Indiana University; UC San Francisco; Department of Defense (retired); University of Southern California; University of California, San Diego; Columbia University Medical Center; Rush University Medical Center; Wien Center; Duke University Medical Center; University of Rochester Medical Center; University of California, Irvine; Medical University South Carolina; Premiere Research Inst (Palm Beach Neurology); University of California, San Francisco; Georgetown University Medical Center; Brigham and Women's Hospital; Banner Sun Health Research Institute; Howard University; University of Wisconsin; University of Washington; Stanford University; Cornell University; ADNI Depression; Principal Investigator; University of California, San Francisco; ATRI PI and Director of Coordinating Center Clinical Core; University of Southern California; University of Southern California; Executive Committee; UC San Francisco; UC San Francisco; University of Southern California; University of Southern California; Mayo Clinic, Rochester; UC Berkeley; Indiana University; University of Southern California; UC Davis; University of Michigan; Data and Publication Committee (DPC); BWH/HMS (Chair); BWM/HMS (Director); Clinical Core Leaders; Core PI; University of Southern California; University of Southern California; University of Southern California; Clinical Informatics, Operations and Regulatory Affairs; USC; USC; USC; USC; USC; USC; USC; Psychiatry Site Leaders and Key Personnel; UC San Francisco; UC San Francisco; UC San Francisco; University of Pittsburgh; University of Pittsburgh; MRI Core Leaders and Key Personnel; Mayo Clinic, Rochester (Core PI); Mayo Clinic, Rochester; Mayo Clinic, Rochester; Mayo Clinic, Rochester; Mayo Clinic, Rochester; Mayo Clinic, Rochester; Mayo Clinic, Rochester; Mayo Clinic, Rochester; PET Core Leaders and Key Personnel; University of Michigan; UC Berkeley; Informatics Core Leaders and Key Personnel; USC (Core PI); USC; USC; Genetics Core Leaders and Key Personnel; Indiana University; Indiana University; Indiana University; Indiana University; Indiana University; University of California, San Francisco: University of Pittsburgh; ^1^Department of Nuclear Medicine, General Hospital of Ningxia Medical University, Yinchuan, China; ^2^The Russell H. Morgan Department of Radiology and Radiological Science, Johns Hopkins University School of Medicine, Baltimore, MD, United States; ^3^Department of Radiology, Xuanwu Hospital of Capital Medical University, Beijing, China; ^4^Center for Reproductive Medicine, General Hospital of Ningxia Medical University, Yinchuan, China; ^5^Mallinckrodt Institute of Radiology, Washington University in St. Louis School of Medicine, St. Louis, MO, United States

**Keywords:** ^18^F-AV1451, Tau PET, cognitively normal, mild cognition impairment, Alzheimer's disease

## Abstract

Recent developments of tau Positron Emission Tomography (PET) allows assessment of regional neurofibrillary tangles (NFTs) deposition in human brain. Among the tau PET molecular probes, ^18^F-AV1451 is characterized by high selectivity for pathologic tau aggregates over amyloid plaques, limited non-specific binding in white and gray matter, and confined off-target binding. The objectives of the study are (1) to quantitatively characterize regional brain tau deposition measured by ^18^F-AV1451 PET in cognitively normal older adults (CN), mild cognitive impairment (MCI), and AD participants; (2) to evaluate the correlations between cerebrospinal fluid (CSF) biomarkers or Mini-Mental State Examination (MMSE) and ^18^F-AV1451 PET standardized uptake value ratio (SUVR); and (3) to evaluate the partial volume effects on ^18^F-AV1451 brain uptake.

**Methods:** The study included total 115 participants (CN = 49, MCI = 58, and AD = 8) from the Alzheimer's Disease Neuroimaging Initiative (ADNI). Preprocessed ^18^F-AV1451 PET images, structural MRIs, and demographic and clinical assessments were downloaded from the ADNI database. A reblurred Van Cittertiteration method was used for voxelwise partial volume correction (PVC) on PET images. Structural MRIs were used for PET spatial normalization and region of interest (ROI) definition in standard space. The parametric images of ^18^F-AV1451 SUVR relative to cerebellum were calculated. The ROI SUVR measurements from PVC and non-PVC SUVR images were compared. The correlation between ROI ^18^F-AV1451 SUVR and the measurements of MMSE, CSF total tau (t-tau), and phosphorylated tau (p-tau) were also assessed.

**Results:** ^18^F-AV1451 prominently specific binding was found in the amygdala, entorhinal cortex, parahippocampus, fusiform, posterior cingulate, temporal, parietal, and frontal brain regions. Most regional SUVRs showed significantly higher uptake of ^18^F-AV1451 in AD than MCI and CN participants. SUVRs of small regions like amygdala, entorhinal cortex and parahippocampus were statistically improved by PVC in all groups (*p* < 0.01). Although there was an increasing tendency of ^18^F-AV-1451 SUVRs in MCI group compared with CN group, no significant difference of ^18^F-AV1451 deposition was found between CN and MCI brains with or without PVC (*p* > 0.05). Declined MMSE score was observed with increasing ^18^F-AV1451 binding in amygdala, entorhinal cortex, parahippocampus, and fusiform. CSF p-tau was positively correlated with ^18^F-AV1451 deposition. PVC improved the results of ^18^F-AV-1451 tau deposition and correlation studies in small brain regions.

**Conclusion:** The typical deposition of ^18^F-AV1451 tau PET imaging in AD brain was found in amygdala, entorhinal cortex, fusiform and parahippocampus, and these regions were strongly associated with cognitive impairment and CSF biomarkers. Although more deposition was observed in MCI group, the ^18^F-AV-1451 PET imaging could not differentiate the MCI patients from CN population. More tau deposition related to decreased MMSE score and increased level of CSF p-tau, especially in ROIs of amygdala, entorhinal cortex and parahippocampus. PVC did improve the results of tau deposition and correlation studies in small brain regions and suggest to be routinely used in ^18^F-AV1451 tau PET quantification.

## Introduction

The neuropathological hallmarks of Alzheimer's disease (AD) are extracellular amyloid-β (Aβ) plaques and the intraneuronal neurofibrillary tangles (NFT), which is primarily composed of hyperphosphorylated tau protein and is a predictor of cognition (1–3). Efforts have been devoted to identifying and developing reliable biomarkers of different stages of AD to differentiate the individuals who would benefit from early intervention (4). It is well-established in AD patients that decreased cerebrospinal fluid (CSF) concentration of the Aβ and increased tau protein could be used as valuable biomarkers (5, 6). CSF total tau (t-tau) and phosphorylated tau (p-tau) improve the sensitivity and specificity of CSF Aβ alone to identify those likely to progress to AD dementia (7–9). Recent researches of tau Positron Emission Tomography (PET) allowed assessment of regional tau deposition in human brain (10–12). *In vivo* imaging of tau pathology is expected to be a useful biomarker in clinical and translational AD researches. Several PET molecular probes, such as ^11^C-PBB3, ^18^F-THK523, ^18^F-THK5105, ^18^F-THK5117, ^18^F-AV68, and ^18^F-AV1451, have recently been radio-synthesized to map the tau distribution in preclinical and clinical studies (13–18).

^18^F-AV1451 is characterized by high selectivity for pathologic tau aggregates over amyloid plaques (17, 19). *n vivo* human studies indicated that patterns of ^18^F-AV1451 retention were paralleled with neuropathological staging of neurofibrillary tau pathology of AD and that tracer retention increased with age even in the presence of cognitive impairment and dementia (18, 20, 21).

It was reported that ^18^F-AV1451 showed off-target binding in caudate, putamen, pallidum and thalamus regions (19, 22, 23). Partial volume correction (PVC) has been introduced and proved effective in improving image quality of tau and amyloid PET as well as accuracy and precision of the quantification analysis (21, 24–26).

In this study, we characterized regional tau deposition by PVC-based ^18^F-AV1451 PET imaging in cognitively normal older adults (CN), mild cognitive impairment (MCI) and AD participants, and evaluated the correlations between CSF biomarkers or Mini-Mental State Examination (MMSE) and ^18^F-AV1451 PET standardized uptake value ratio (SUVR). For comparison purpose, analysis on the PET without PVC were also included in this study.

## Materials and Methods

Data used in this article were obtained from the ADNI database (adni.loni.usc.edu). The ADNI was launched in 2003 as a public-private partner-ship by the National Institute on Aging, the Food and Drug Administration, private pharmaceutical companies and non-profit organizations. Its primary goal was to test whether neuroimaging like serial magnetic resonance imaging (MRI), PET, biological markers, and clinical and neuropsychological assessment can be combined to measure the progression of MCI and early AD (27). A detailed description of the inclusion criteria can be found on the ADNI website (www.adni-info.org). Data were downloaded from the ADNI database (adni.loni.usc.edu). All participants signed written informed consent for participation in the ADNI, as approved by the institutional board at each participating center.

### Participants

One hundred and fifteen participants with ^18^F-AV1451 PET, T1-weighted magnetization-prepared rapid-acquisition gradient-echo (MP-RAGE) or inversion recovery spoiled gradient-echo (IR-SPGR) MRI were included from ADNI 1, 2. and GO database. The complete list of exclusion and inclusion criteria of ADNI can be found online (https://adni.loni.usc.edu/wp-content/uploads/2008/07/adni2-procedures-manual.pdf) Pre-processed ^18^F-AV1451 PET brain images and corresponding T1-weighted MP-RAGE MRI images were downloaded from the ADNI database in March 2017. The last known diagnostic status was the one mentioned at the time of the last visit listed in the dataset. Their clinical diagnosis was cognitively normal (CN) (*N* = 49), MCI (*N* = 58), and AD (*N* = 8). Demographics, CSF biomarkers and clinical assessments were also downloaded from ADNI database.

### PET Acquisition

The radiochemical synthesis of ^18^F-AV1451 was overseen and regulated by Avid Radiopharmaceuticals and distributed to the qualifying ADNI sites. PET imaging was performed at each ADNI site according to standardized protocols. Eighty minutes post-injection of about 10 mCi of ^18^F-AV1451 followed by the acquisition. Data were collected as 5 min per frame from 80 to 100 min post-tracer injection. PET with computed tomography imaging (PET/CT) scans preceded these acquisitions with a CT scan for attenuation correction; PET-only scanners performed a transmission scan following the emission scan.

### MRI Acquisition

Structural MRIs were acquired at ADNI sites. All participants had 1.5-T or 3.0-T MRI scan with a three-dimensional (3D) MP-RAGE or IR-SPGR T1-weighted sequences with sagittal slices and voxel size of 1.1 × 1.1 × 1.2 mm^3^. The full details were described in online manual (http://adni.loni.usc.edu/methods/documents/mri-protocols).

### PET Processing and Quantification

T1-weighted MRI and pre-processed PET images were downloaded from ADNI. All downloaded PET images were pre-processed to have standard orientation, same image volume size (160 × 160 × 96 in x, y, z) and voxel size (1.5 × 1.5 × 1.5 mm in x, y, z), spatial resolution of 8 mm in full width at half maximum (FWHM) in x, y, z, respectively, by ADNI (28). Statistical Parametric Mapping software (SPM8, Wellcome Department of Imaging Neuroscience, London, United Kingdom) and MATLAB (The MathWorks Inc.) were used for further processing of the downloaded PET and MRI images. All pre-processed mean PET images were coregistered to structure MRI images. MRI images were normalized to standard Montreal Neurologic Institute (MNI) space using SPM8 with an MRI template provided by VBM8 toolbox (29, 30). PET-MRI image processing including PET-to-MRI coregistration was described in our previous study (31). PVC was employed to account for partial volume effects due to brain atrophy and signal spillover. A reblurred Van Cittertiteration method (32, 33) was used for PVC on the mean images. For the PVC method, a 3-D Gaussian kernel of 8 mm FWHM was used for spatial smoothing function h, step length α = 1.5, and the iteration was stopped if relative percent change of PVC images <1% (33). Both PVC and non-PVC mean images were coregistered to structure MRI image for MRI-based spatial normalization. A total of 26 region of interests (ROIs) were defined on the MRI template using PMOD software (PMOD Technologies Ltd., Zürich, Switzerland) in the standard MNI space, and the ROI SUVRs were calculated by using cerebellum as reference tissue with and without PVC. A global cortex was defined as a combination of orbital frontal, prefrontal, superior frontal, lateral temporal, parietal, posterior precuneus, occipital, anterior cingulate, and posterior cingulate.

### Clinical and Cognitive Assessments

All participants were assessed with a wide spectrum of clinical and cognitive tests (34). In this study, we used the global Clinical Dementia Rating (CDR), the MMSE score, Alzheimer's Disease Assessment Scale-cognitive subscale (ADAS-cog 11) and ADAS-cog 13. Neuropsychiatric symptoms were assessed by the Neuropsychiatric Inventory Questionnaire (NPI-Q). The depressive features and functional ability were assessed with the Geriatric Depression Scale-Short Form (GDS) and Functional Assessment Questionnaire (FAQ).

### CSF Biomarkers Assessment

The studied variables of CSF biomarkers were Aβ, t-tau, p-tau, the tau/Aβ, and p-tau/Aβ ratio. Apolipoprotein E (ApoE) gene typing was carried out on all participants during the first visit. CSF t-tau, p-tau, and Aβ42 were collected from all participants and measured using the multiplex xMAP Luminex platform (Luminex Corp., Austin, TX, USA) with the INNO-BIA AlzBio3 kit (Innogenetics, Ghent, Belgium) (6, 35). Full details on the collection, processing, storage, analysis, and quality control procedures for CSF samples can be found online (http://adni.loni.usc.edu/methods/documents/).

### Statistical Analyses

Continuous variables were presented as mean ± SD and categorical variables as number (percent). The characteristics of subjects were compared among CN, MCI, and AD groups using generalized linear model (GLM) for continuous variables and Chi-squared analysis for categorical variables. GLM was employed in the comparison of SUVRs among CN, MCI, and AD groups adjusted with age and education levels. The correlations between SUVR and MMSE score, SUVR and CSF tau biomarkers were evaluated using linear regression. SUVRs differences between non-PVC and PVC were assessed by paired student's *t*-test. A two-sided *p*-value < 0.05 was considered statistically significant. All statistical analysis was performed using statistical package of social science (SPSS) version 24.0 software.

## Results

### Demographics and Clinical Characteristics

Diagnostic grouping was based on the diagnosis evaluation provided by the ADNI clinical Core. We ended up with 49 CN, 58 MCI, and 8 AD patients. The participants' demographics, ApoE type 4 allele (ApoE ε4) status, cognitive scores and CSF biomarkers are detailed in Table 1.

**Table 1:** Demographics and clinical characteristics.

**Characteristics**	**CN**	**MCI**	**AD**	**F/χ^2^**	***P-*****value**		
	**(*N* = 49)**	**(*N* = 58)**	**(*N* = 8)**		**CN-MCI**	**MCI-AD**	**CN-AD**
Sex (F/M)	24/25	16/42	5/3	7.08	0.02	0.05	0.48
Age, year	74.79 ± 6.68	77.95 ± 7.50	76.83 ± 8.74	2.53	0.07	0.91	0.74
ApoE ε4 (–/+)	30/19	38/20	2/6	4.85	0.65	0.03	0.06
Education, year	16.33 ± 2.23	16.67 ± 2.88	15.00 ± 3.02	1.46	0.78	0.22	0.39
Aβ, pg/ml	1204.80 ± 458.64	1125.76 ± 473.46	771.35 ± 200.16	2.76	0.58	0.06	0.02
t-tau, pg/ml	243.89 ± 100.53	265.77 ± 125.09	401.36 ± 123.06	5.68	0.52	0.002	<0.001
p-tau, pg/ml	22.54 ± 10.89	25.04 ± 12.33	38.89 ± 13.01	5.82	0.44	0.002	<0.001
t-tau/Aβ	0.24 ± 0.14	0.28 ± 0.19	0.55 ± 0.19	10.50	0.23	<0.001	<0.001
p-tau/Aβ	0.02 ± 0.01	0.03 ± 0.02	0.05 ± 0.02	8.17	0.14	<0.001	<0.001
ADAS_cog11	4.96 ± 2.48	9.00 ± 4.39	21.88 ± 9.66	56.08	<0.001	<0.001	<0.001
ADAS_cog13	7.76 ± 4.41	14.31 ± 6.58	33.63 ± 11.50	62.69	<0.001	<0.001	<0.001
Global CDR	0.07 ± 0.18	0.40 ± 0.26	1.06 ± 0.62	51.96	<0.001	<0.001	<0.001
NPI-Q	0.92 ± 2.52	1.55 ± 2.66	6.75 ± 4.20	15.76	0.43	<0.001	<0.001
MMSE	28.63 ± 1.82	27.66 ± 2.16	20.38 ± 4.53	46.30	0.07	<0.001	<0.001
FAQ	0.40 ± 1.76	3.18 ± 5.07	13.63 ± 9.91	29.57	0.01	<0.001	<0.001
GDScale	1.02 ± 1.18	2.03 ± 2.07	3.13 ± 1.55	7.28	0.01	0.21	0.004

The age and education years of all the participants in this study were (76.52 ± 7.34) and (16.41 ± 2.64). No significant differences in age and education levels were found among CN, MCI, and AD groups. Moderate differences were found in the proportion of sex between CN and MCI groups, and the proportion of ApoE ε4 between MCI and AD groups, respectively. Compared with CN and MCI participants, AD patients were more impaired on MMSE score and performed higher levels on CSF t-tau, p-tau, t-tau/ Aβ, p-tau/ Aβ, ADAS-cog 11, ADAS-cog 13, Global CDR, NPI-Q, and FAQ scores (*p* < 0.01). There was no significant difference on levels of all CSF biomarkers between CN and MCI group (all *p* > 0.05).

### Brain Tau Deposition in CN, MCI, and AD Participants

**Figure 1:**
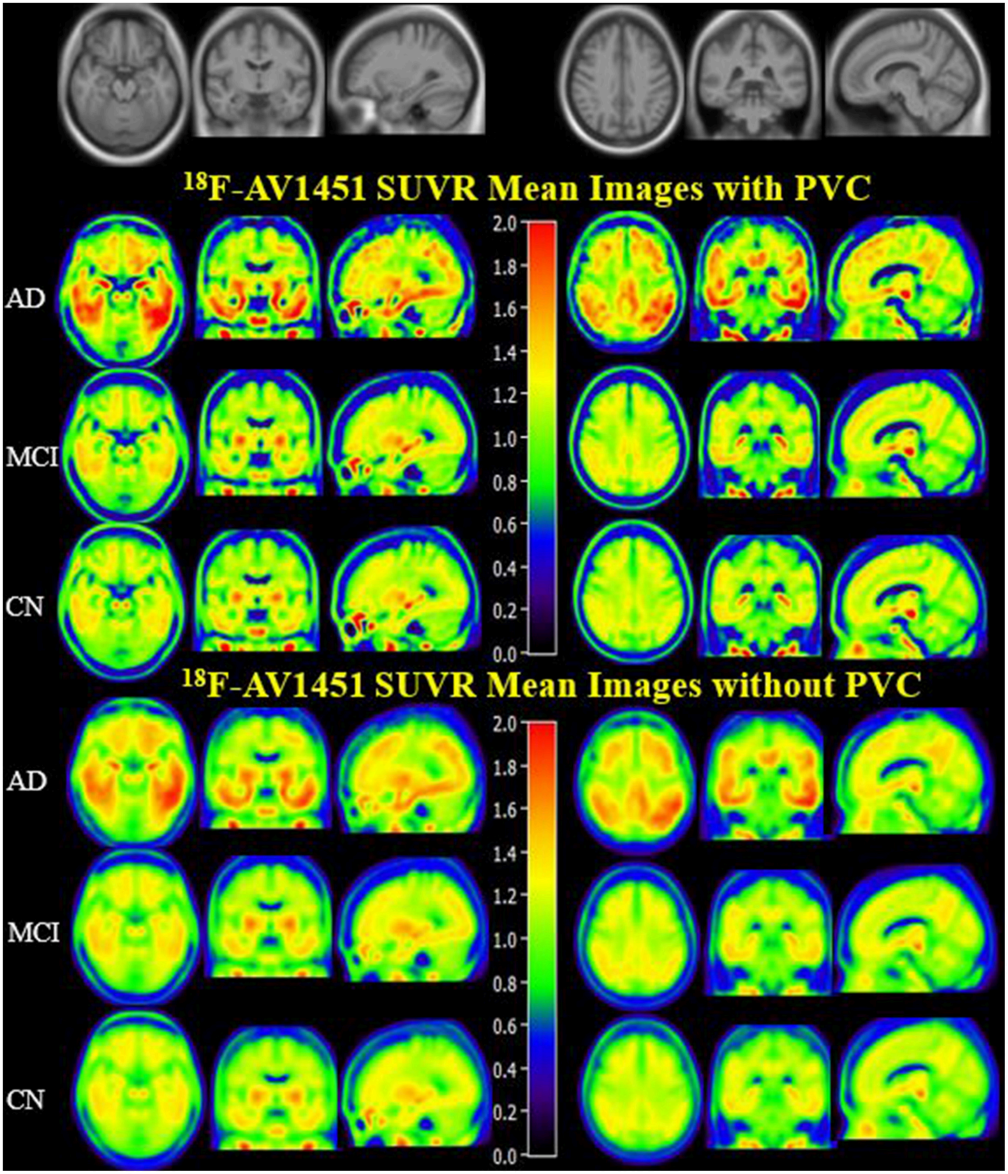
^18^F-AV1451 SUVR Mean Images with PVC and non-PVC. Mean images generated by computing the mean of images from AD, MCI, and CN participants separately. The upper images have been corrected with PVC, whereas the lower images without PVC.

PVC for this study was used to decrease off-target binding in choroid plexus close to the hippocampus, basal ganglia, and brain atrophy. In order to create an anatomical visualization of the effects of PVC on spatial resolution and image contrast, we displayed mean ^18^F-AV1451 SUVR images of CN, MCI, and AD with and without PVC in Figure 1, which clearly showed an increased image spatial resolution and contrast after PVC in most cortical and subcortical ROIs. Brain ^18^F-AV1451 SUVR patterns were visually consistent with previous post-mortem and *in vivo* PET studies (1, 20, 21).

**Figure 2:**
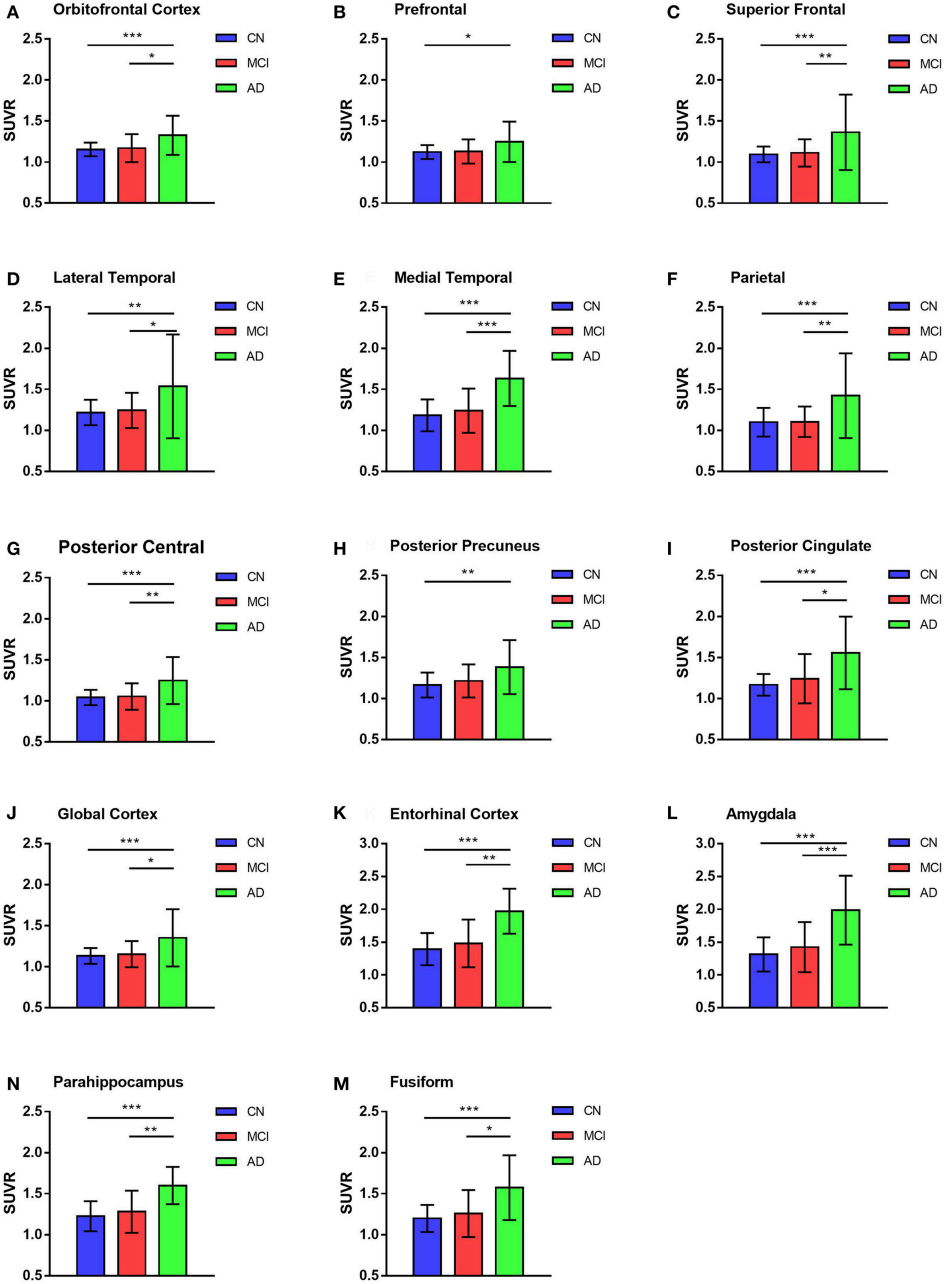
^18^F-AV1451 SUVRs of cortical and subcortical ROIs in AD, MCI, and CN groups. Bar graphs showing ROIs SUVR (mean with error bars depicting SD) comparison among AD, MCI and CN groups in the orbitofrontal cortex **(A)**, prefrontal cortex **(B)**, superior frontal cortex **(C)**, lateral temporal cortex **(D)**, medial temporal cortex **(E)**, parietal cortex (F), posterior central cortex **(G)**, posterior precuneus **(H)**, posterior cingulate **(I)**, global cortex **(J)**, entorhinal cortex **(K)**, amygdala **(L)**, parahippocampus **(N)** and fusiform **(M)**. *P*-value was defined using GLM to compare SUVR between CN and MCI, CN and AD, MCI and AD. *p < 0.05, **p < 0.01, ***p < 0.001.

GLM was used in the comparison of SUVRs of ROIs between and among groups and adjusted with age and education levels. The highest deposition regions of ^18^F-AV1451 among the 26 ROIs were amygdala, entorhinal cortex, parahippocampus, fusiform, and posterior cingulate. More deposition was observed in parietal, posterior precuneus and frontal cortex, whereas less concentration in hippocampus and occipital brain regions. For AD participants, there were significantly more ^18^F-AV1451 retention in amygdala, entorhinal cortex, parahippocampus, superior frontal cortex, medial temporal cortex, and parietal cortex than CN and MCI participants (all *p* < 0.01), which was shown in Figure 2. The average SUVRs of entorhinal cortex, amygdala and parahippocampus in CN group were 1.27, 1.26, and 1.18, respectively. In comparison, they were 1.32, 1.33, and 1.21 in MCI group, respectively. Increased SUVRs were observed with PVC studied. Although there was an increasing tendency of SUVRs in MCI group, no statistical different was found in all ROIs between CN and MCI participants with or without PVC (*p* > 0.05).

**Figure 3:**
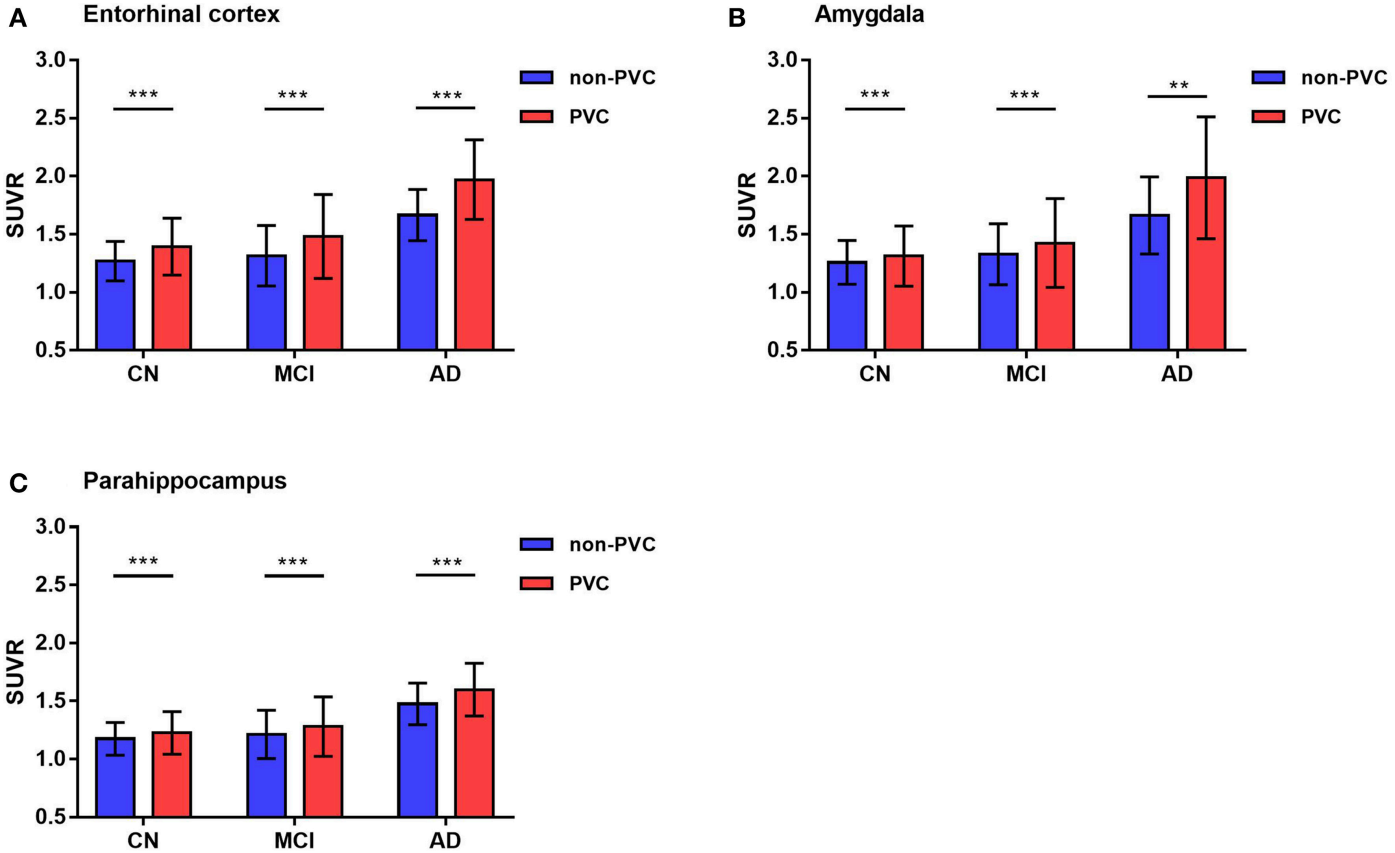
Representative ^18^F-AV1451 SUVRs comparison with or without PVC. Bar graphs showing ROIs SUVR (mean with error bars depicting SD) comparison between PVC and non-PVC in entorhinal cortex **(A)**, amygdala **(B)** and parahippocampus **(C)**. *P*-value was defined using paired *t*-test to compare SUVR between PVC and non-PVC images in CN, MCI, and AD groups. **p < 0.01, ***p < 0.001.

Notably, these results were observed in both PVC and non-PVC studies. The entorhinal cortex SUVR in AD group with or without PVC was 1.97 vs. 1.69, which showed a 18% promotion in quantitative analysis. In regions of amygdala, PVC improved almost 20% of the SUVR. These results were observed in many small regions not only in AD group but also in MCI and CN group. Using paired student's *t*-test, 22 of the 26 ROIs showed statistical difference with or without PVC. Of note, SUVRs of small regions like amygdala, entorhinal cortex, and parahippocampus were statistically improved by PVC in all groups (*p* < 0.01), which were shown in Figure 3.

### Correlations Between Regional ^18^F-AV1451 SUVRs and MMSE Score, and Between ^18^F-AV1451 SUVRs and CSF Tau Biomarkers

**Figure 4:**
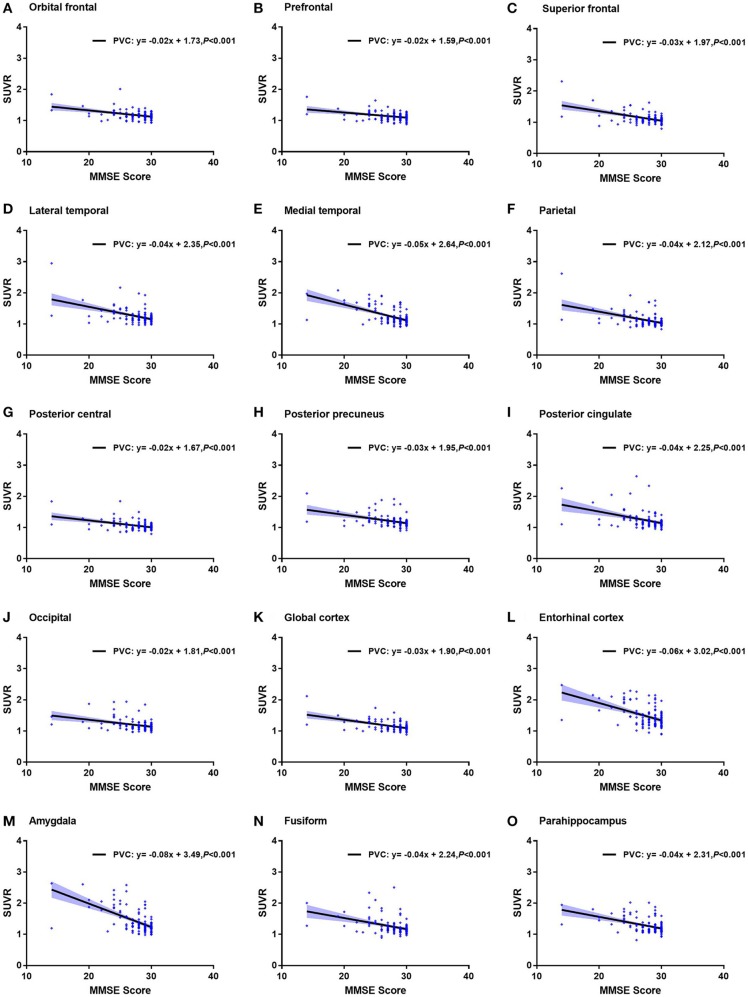
Correlation of MMSE and ^18^F-AV1451 deposition in ROI SUVRs with PVC. Correlation of MMSE and ROI SUVRs with PVC in the orbitofrontal cortex **(A)**, prefrontal cortex **(B)**, superior frontal cortex **(C)**, lateral temporal cortex **(D)**, medial temporal cortex **(E)**, parietal cortex **(F)**, posterior central cortex **(G)**, posterior precuneus **(H)**, posterior cingulate **(I)**, occipital cortex **(J)**, global cortex **(K)**, entorhinal cortex **(L)**, amygdala **(M)**, fusiform **(N)** and parahippocampus **(O)**.

We found that MMSE score, CSF t-tau, and p-tau in 15 ROIs were significantly associated with the ROI-based ^18^F-AV-1451 SUVR (*p* < 0.05). Increasing ^18^F-AV1451 retention was significantly related with decreasing MMSE score and increasing CSF p-tau. The correlations of MMSE and the ROI SUVRs with PVC were shown in Figure 4.

**Figure 5:**
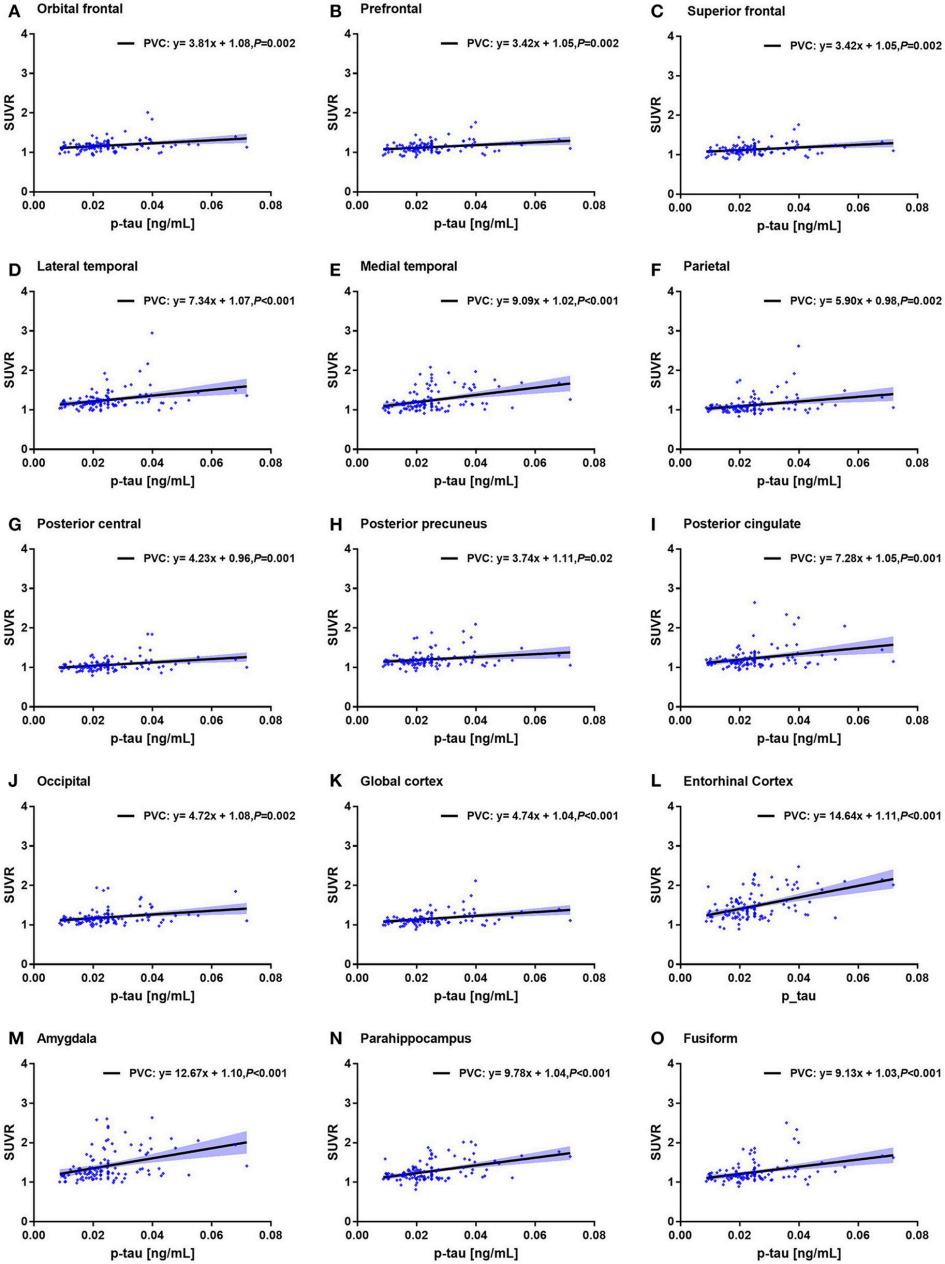
Correlation of CSF p-tau level and ^18^F-AV1451 deposition in ROI SUVRs with PVC. Correlation of CSF p-tau level and ROI SUVRs with PVC in the orbitofrontal cortex **(A)**, prefrontal cortex **(B)**, superior frontal cortex **(C)**, lateral temporal cortex **(D)**, medial temporal cortex **(E)**, parietal cortex **(F)**, posterior central cortex **(G)**, posterior precuneus **(H)**, posterior cingulate **(I)**, occipital cortex (J), global cortex **(K)**, entorhinal cortex **(L)**, amygdala **(M)**, parahippocampus **(N)** and fusiform **(O)**.

For example, the correlation coefficient between amygdala SUVR and MMSE score was −0.60, which was statistically different (*p* < 0.001). The correlation coefficient between entorhinal cortex SUVR and CSF p-tau value was 0.48, which was also statistically different (*p* < 0.001). Notably, the correlation between ROI SUVRs and MMSE score and between ROI SUVRs and CSF tau biomarkers were observed in both PVC and non-PVC studies. Similar with deposition study of ^18^F-AV-1451, PVC improved the correlation study, especially in small brain regions. The correlations of CSF p-tau and the ROI SUVRs with PVC were shown in Figure 5.

## Discussion

In this study, we summarized the deposition pattern of ^18^F-AV1451 in CN, MCI, and AD brains and assessed the ROI SUVRs of ^18^F-AV1451 association with clinical cognitive measures and CSF biomarkers in CN, MCI, and AD participants. PVC was used as a routine method and age and education level were also included as covariates in this study.

The main finding of this study was that although there was an increasing tendency of ^18^F-AV1451 SUVRs in MCI group compared with CN group, no significant difference of ^18^F-AV1451 deposition was found between CN and MCI brains with or without PVC. We confirmed that more tau deposition related to decreased MMSE score and increased level of CSF p-tau, especially in ROIs of amygdala, entorhinal cortex, and parahippocampus. PVC improved the results of ^18^F-AV1451 tau deposition and correlation studies in small brain regions.

The differences among CN, MCI, and AD participants were highly significant for ApoE ε4 status, cognition and four CSF markers, except for Aβ level. It is widely reported that ApoE ε4 genotype is one of the major risk factors in AD. In this study, 75% of AD participants were ApoE ε4 carriers. Also, we got the similar result that the females are more likely to develop AD compared with the males, and education years are not declined with the cognitive function as reported (36, 37). The level of CSF t-tau and p-tau could reflect the tau deposition in brain to some extent. We also found that CSF t-tau and p-tau levels in AD participants were significantly higher than MCI and CN participants.

One of the earliest regions to degenerate to AD and the first tau deposition region is the mesial temporal lobe (1). Because of the retention in temporal lobe in normal aging adults as reported (21), we included age and education level as covariant for comparison of ROI ^18^F-AV1451 SUVRs among different groups. Our major findings in ROI-based SUVRs were as follows: increased ^18^F-AV1451 prominently deposit in entorhinal cortex, amygdala, parahippocampus, fusiform, as well as posterior cingulate and temporal brain region, followed by parietal and frontal brain regions, and to a less degree in the hippocampus and occipital brain regions in the full cohort, which were similar in previous reports (38). Scholl et al. focused on the uptake of ^18^F-AV1451 in participants with different PiB status and found localized and increased uptake of ^18^F-AV1451 in temporal lobe regions, medial temporal subregions and partly in inferior and lateral temporal cortical regions in PiB-negative old healthy adults. AD patients showed higher retention of ^18^F-AV1451 particularly throughout temporal lobe to encompass a larger region of parietal and frontal cortex (21). Cho et al. reported ^18^F-AV1451 tau PET accumulation most frequently observed in the medial temporal regions and stepwise spread to basal and lateral temporal, inferior parietal, posterior cingulate, and the other association cortices (39). They also found tau accumulation was most frequently deposited in the entorhinal cortex and parahippocampal cortex, then involved in the fusiform, inferior temporal cortex, amygdala, middle temporal, inferior parietal, posterior cingulate. Like postmortem and other different cohorts' study (2, 40), the entorhinal cortex was the most frequently involved region followed by adjacent limbic areas (amygdala and parahippocampus) in our study.

Johnson et al. found a higher ^18^F-AV1451 binding in MCI/AD group compared with CN group in entorhinal cortex and parahippocampus (20). In this study we compared the difference of SUVRs between CN and MCI participants. Of all the ROIs, none of the SUVRs was statistically different between CN and MCI in our study. However, these regions can be easily distinguished: orbital frontal, prefrontal, superior frontal, lateral temporal, medial temporal, parietal, posterior precuneus, posterior cingulate, global cortex, amygdala, entorhinal, fusiform, and parahippocampus. Although there was no significant difference between MCI and CN group, there was still a clear increasing trend for tau binding. For example, SUVR in entorhinal cortex was 1.39 in CN, but it was 1.48 in MCI brain. When compared with CN brain, SUVRs in MCI brain increased by 2–9%.

It was reported that PVC has improved the tau PET quantification (23, 41). To deal with off-target binding and brain atrophy on PET quantification, we performed the PVC routinely in this study. In CN group, 85% ROIs were statistically different with or without PVC. However, the percentage fell to 73 and 35% in MCI and AD group, respectively. PVC showed better adjustment of SUVRs, especially in CN group. As visualized directly in ^18^F-AV1451 SUVR mean image, PVC improved the spatial resolution and image contrast. The amygdala SUVR with or without PCR in AD brain was 1.66 and 1.99, which almost increased by 20% after PVC. In comparison of SUVRs of posterior precuneus region between MCI and AD group, the statistical status was evenly changed.

In all participants, SUVRs of substantia nigra, ventral striatum and putamen were at a higher level, but no statistical differences among groups were observed. This could be due to the off-target binding of ^18^F-AV1451, brain atrophy and tau accumulation in neurodegeneration. Hippocampal ^18^F-AV1451 SUVR in all three groups were more than 1.2. However, there was no significant increase in the AD progression in this study, and this will be studied and verified by a large population, especially more AD patients enrolled, with going ADNI project.

We also observed a significant relationship between MMSE score and ^18^F-AV1451 SUVR. In this study, declined MMSE score was observed with increasing ^18^F-AV1451 binding in amygdala, entorhinal cortex, parahippocampus, and fusiform as shown in Figure 4, which was paralleled with cognitive impairment and AD progression. Besides cognitive status, we also found significant association between the ROI SUVRs and CSF t-tau and p-tau level, which was similar to the previous reports (20). Mattsson et al. evaluated the performance of ^18^F-AV1451 PET imaging and CSF tau in different clinical stages of AD and found that ^18^F-AV1451 exhibited closely to perfect diagnosis in mild and moderate AD (42). As CSF p-tau is a biomarker of AD, we also observed a positive correlation between CSF p-tau level and ^18^F-AV1451 deposition as seen in Figure 5. Ascending ^18^F-AV1451 deposition in cortical and subcortical ROIs, especially in amygdala, entorhinal cortex, fusiform, and parahippocampus, were observed with more pathological tau deposition in brain. The correlation results of MMSE and regional ^18^F-AV1451 binding was partially consistent with previous study, which evaluated the correlation between inferior temporal ^18^F-AV1451 binding and MMSE in all participants, CN and MCI/AD group (20). The rho value in all participants was −0.46. In this study the rho value of amygdala ^18^F-AV1451 SUVR and MMSE score was −0.60 (*p* < 0.001, Figure 4M), and the rho value of entorhinal cortex ^18^F-AV1451 binding and CSF p-tau level was 0.48 (*p* < 0.001, Figure 5L). We routinely applied PVC to account for off-target binding and atrophy effects. When we examined correlations between non-PVC SUVRs and MMSE score, although correlations were weaker, the overall pattern of results were consistent.

## Conclusions

The typical deposition of ^18^F-AV1451 tau PET imaging in AD brain was found in amygdala, entorhinal cortex, fusiform and parahippocampus, and these regions were strongly associated with cognitive impairment and CSF biomarkers. Although more deposition was observed in MCI group, the ^18^F-AV-1451 PET imaging could not differentiate the MCI patients from CN population. More tau deposition related to decreased MMSE score and increased level of CSF p-tau, especially in ROIs of amygdala, entorhinal cortex, and parahippocampus. PVC did improve the results of tau deposition and correlation studies in small brain regions and is suggested to be routinely used in ^18^F-AV1451 tau PET quantification.

## Ethics Statement

Data used in the preparation of this article were obtained from the ADNI database (adni.loni.usc.edu). The ADNI was launched in 2003 as a public-private partner-ship by the National Institute on Aging, the Food and Drug Administration, private pharmaceutical companies, and non-profit organizations. Its primary goal was to test whether neuroimaging like serial MRI, PET, biological markers, and clinical and neuropsychological assessment can be combined to measure the progression of MCI and early AD. ADNI is the result of efforts of multicenter project with over 50 medical centers and university sites across the USA and Canada (29). A detailed description of the inclusion criteria can be found on the ADNI website (www.adni-info.org). Data were downloaded from the ADNI database (adni.loni.usc.edu) and included all participants. All participants signed written informed consent for participation in the ADNI, as approved by the institutional board at each participating center.

## Author Contributions

YZ study concept and design. QZ, LH, ML, and YZ data collecting and analysis. QZ and YZ drafting and editing the manuscript. QZ and ML statistics and figures. The investigators within the ADNI contributed to the design and implementation of ADNI and/or provided data but did not participate in analysis or writing of this report.

### Conflict of Interest Statement

The authors declare that the research was conducted in the absence of any commercial or financial relationships that could be construed as a potential conflict of interest.

## References

[1] Braak H Alafuzoff I Arzberger T Kretzschmar H Del Tredici K. Staging of Alzheimer disease-associated neurofibrillary pathology using paraffin sections and immunocytochemistry. Acta Neuropathol. (2006) 112:389–404. 10.1007/s00401-006-0127-z 16906426 PMC3906709

[2] Braak H Braak E Neuropathological staging of Alzheimer-related changes. Acta Neuropathol. (1991) 82:239–59. 1759558 10.1007/BF00308809

[3] Nelson PT Alafuzoff I Bigio EH Bouras C Braak H Cairns NJ. Correlation of Alzheimer disease neuropathologic changes with cognitive status: a review of the literature. J NeuropatholExp Neurol. (2012) 71:362–81. 10.1097/NEN.0b013e31825018f7 22487856 PMC3560290

[4] Jack CR Knopman DS Jagust WJ Shaw LM Aisen PS Weiner MW. Hypothetical model of dynamic biomarkers of the Alzheimer's pathological cascade. Lancet Neurol. (2010) 9:119–28. 10.1016/S1474-4422(09)70299-6 20083042 PMC2819840

[5] Sunderland T Linker G Mirza N Putnam KT Friedman DL Kimmel LH. Decreased β-amyloid1–42 and increased tau levels in cerebrospinal fluid of patients with Alzheimer disease. JAMA. (2003) 289:2094–103. 10.1001/jama.289.16.2094 12709467

[6] Shaw LM Vanderstichele H Knapik-Czajka M Clark CM Aisen PS Petersen RC. Cerebrospinal fluid biomarker signature in Alzheimer's Disease Neuroimaging Initiative subjects. Ann Neurol. (2009) 65:403–13. 10.1002/ana.21610 19296504 PMC2696350

[7] Andreasen N Minthon L Davidsson P Vanmechelen E Vanderstichele H Winblad B. Evaluation of CSF-tau and CSF-Abeta42 as diagnostic markers for Alzheimer disease in clinical practice. Arch Neurol. (2001) 58:373–9. 11255440 10.1001/archneur.58.3.373

[8] Schoonenboom NS Pijnenburg YA Mulder C Rosso SM Van Elk EJ Van Kamp GJ. Amyloid beta (1-42) and phosphorylated tau in CSF as markers for early-onset Alzheimer disease. Neurology. (2004) 62:1580–4. 15136685 10.1212/01.wnl.0000123249.58898.e0

[9] Roe CM Fagan AM Grant EA Hassenstab J Moulder KL Maue Dreyfus D. Amyloid imaging and CSF biomarkers in predicting cognitive impairment up to 7.5 years later. Neurology. (2013) 80:1784–91. 10.1212/WNL.0b013e3182918ca6 23576620 PMC3719431

[10] Dani M Brooks DJ Edison P. Tau imaging in neurodegenerative diseases. Eur Nucl Med Mol Imaging. (2016) 43:1139–50. 10.1007/s00259-015-3231-2 26572762 PMC4844651

[11] Saint-Aubert L Lemoine L Chiotis K Leuzy A Rodriguez-Vieitez E Nordberg A. Tau PET imaging: present and future directions. Mol Neurodegener. (2017) 12:19. 10.1186/s13024-017-0162-3 28219440 PMC5319037

[12] Villemagne VL Fodero-Tavoletti MT Masters CL Rowe CC. Tau imaging: early progress and future directions. Lancet Neurol. (2015) 14:114–24. 10.1016/S1474-4422(14)70252-2 25496902

[13] Wood H. Alzheimer disease: [11C] PBB3: a new PET ligand that identifies tau pathology in the brains of patients with AD. Nat Rev Neurol. (2013) 9:599. 10.1038/nrneurol.2013.216 24145369

[14] Okamura N Furumoto S Harada R Tago T Yoshikawa T Fodero-Tavoletti M. Novel 18F-labeled arylquinoline derivatives for noninvasive imaging of tau pathology in Alzheimer disease. J Nucl Med. (2013) 54:1420–7. 10.2967/jnumed.112.117341 23857514

[15] Kamura N Furumoto S Fodero-Tavoletti M Mulligan RS Harada R Yates P Non-invasive assessment of Alzheimer's disease neurofibrillary pathology using 18F-THK-5105 PET. Brain. (2014) 137:1762–71. 10.1093/brain/awu064 24681664

[16] Chien DT Szardenings AK Bahri S Walsh JC Mu F Xia C. Early clinical PET imaging results with the novel PHF-tau radioligand [F18]-T808. J Alzheimers Dis. (2014) 38:171–84. 10.3233/JAD-130098 23948934

[17] Xia CF Arteaga J Chen G Gangadharmath U Gomez LF Kasi D [18F]-T807, a novel tau positron emission tomography imaging agent for Alzheimer's disease. Alzheimers Dement. (2013) 9:666–76. 10.1016/j.jalz.2012.11.008 23411393

[18] Chien DT Bahri S Szardenings AK Walsh JC Mu F Su MY. Early clinical PET imaging results with the novel PHF-tau radioligand [F-18]-T807. J Alzheimers Dis. (2013) 34:457–68. 10.3233/JAD-122059 23234879

[19] Marquié M Normandin MD Vanderburg CR Costantino IM Bien EA Klunk WE. Validating novel tau positron emission tomography tracer [F-18]-AV-1451 (T807) on postmortem brain tissue. Ann Neurol. (2015) 78:787–800. 10.1002/ana.24517 26344059 PMC4900162

[20] Johnson KA Schultz A Betensky RA Becker JA Sepulcre J Rentz D. Tau positron emission tomographic imaging in aging and early Alzheimer disease. Ann Neurol. (2016) 79:110–9. 10.1002/ana.24546 26505746 PMC4738026

[21] Scholl M Lockhart SN Schonhaut DR O'Neil JP Janabi M Ossenkoppele R. PET imaging of tau deposition in the aging human brain. Neuron. (2016) 89:971–82. 10.1016/j.neuron.2016.01.028 26938442 PMC4779187

[22] Marquié M Normandin MD Meltzer AC Siao Tick Chong M Andrea NV Antón-Fernández A Pathological correlations of [F-18]-AV-1451 imaging in non-alzheimer tauopathies. Ann. Neurol. (2017) 81:117–28. 10.1002/ana.24844 27997036 PMC5319193

[23] Baker SL Maass A Jagust WJ. Considerations and code for partial volume correcting [18F]-AV-1451 tau PET data. Data Brief. (2017) 15:648–57. 10.1016/j.dib.2017.10.024 29124088 PMC5671473

[24] Su Y Blazey TM Snyder AZ Raichle ME Marcus DS Ances BM. Partial volume correction in quantitative amyloid imaging. Neuroimage. (2015) 107:55–64. 10.1016/j.neuroimage.2014.11.058 25485714 PMC4300252

[25] Matsubara K Ibaraki M Shimada H Ikoma Y Suhara T Kinoshita T Impact of spillover from white matter by partial volume effect on quantification of amyloid deposition with [11C] PiB PET. Neuroimage. (2016) 143:316–24. 10.1016/j.neuroimage.2016.09.028 27639351

[26] Brendel M Högenauer M Delker A Sauerbeck J Bartenstein P Seibyl J. Improved longitudinal [(18)F]-AV45 amyloid PET by white matter reference and VOI-based partial volume effect correction. Neuroimage. (2015) 108:450–9. 10.1016/j.neuroimage.2014.11.055 25482269

[27] Petersen RC Aisen PS Beckett LA Donohue MC Gamst AC Harvey DJ. Alzheimer's Disease Neuroimaging Initiative (ADNI): clinical characterization. Neurology. (2010) 74:201–9. 10.1212/WNL.0b013e3181cb3e25 20042704 PMC2809036

[28] Drzezga A Riemenschneider M Strassner B Grimmer T Peller M Knoll A. Cerebral glucose metabolism in patients with AD and different ApoE genotypes. Neurology. (2005) 64:102–7. 10.1212/01.WNL.0000148478.39691.D3 15642911

[29] Ashburner J Friston KJ. Unified segmentation. Neuroimage. (2005) 26:839–51. 10.1016/j.neuroimage.2005.02.018 15955494

[30] Gaser C Voxel Based Morphometry Extension to SPM8. Available online at: http://www.neuro.uni-jena.de/vbm/ 2014/

[31] Chen X Zhou Y Wang R Cao H Reid S Gao R. Potential clinical value of multiparametric PET in the prediction of Alzheimer's disease progression. PLoS ONE. (2016) 11:e0154406. 10.1371/journal.pone.0154406 27183116 PMC4868310

[32] Tohka J Reilhac A. Deconvolution-based partial volume correction in Raclopride-PET and Monte Carlo comparison to MR-based method. Neuroimage. (2008) 39:1570–84. 10.1016/j.neuroimage.2007.10.038 18077187

[33] Paranjpe MD Chen X Liu M Paranjpe I Leal JP Wang R The effects of ApoE ε4 on longitudinal brain region-specific glucose metabolism in patients with mild cognitive impairment: a FDG-PET study. Neuroimage. (2019) 22:101795 10.1016/j.nicl.2019.101795 30991617 PMC6449776

[34] Aisen PS Petersen RC Donohue MC Gamst A Raman R Thomas RG. Clinical core of the Alzheimer's disease neuroimaging initiative: progress and plans. Alzheimers Dement. (2010) 6: 239–46. 10.1016/j.jalz.2010.03.006 20451872 PMC2867843

[35] Olsson A Vanderstichele H Andreasen N De Meyer G Wallin A Holmberg B Simultaneous measurement of β-amyloid1–42, total tau, and phosphorylated tau (Thr181) in cerebrospinal fluid by the xMAP technology. Clin Chem. (2005) 51:336–45. 10.1373/clinchem.2004.039347 15563479

[36] Damoiseaux JS Seeley WW Zhou J Shirer WR Coppola G Karydas A. Gender modulates the ApoE epsilon4 effect in healthy older adults: convergent evidence from functional brain connectivity and spinal fluid tau levels. J Neurosci. 2012) 32:8254–62. 10.1523/JNEUROSCI.0305-12.2012 22699906 PMC3394933

[37] Stern Y. Cognitive reserve in ageing and Alzheimer's disease. Lancet Neurol. (2012) 11:1006–12. 10.1016/S1474-4422(12)70191-6 23079557 PMC3507991

[38] Schwarz AJ Yu P Miller BB Shcherbinin S Dickson J Navitsky M Regional profiles of the candidate tau pet ligand 18F-av1451 recapitulate key features of braak histopathological stage. Brain. (2016) 139:1539–50. 10.1093/brain/aww023 26936940

[39] Cho H Choi JY Hwang MS Kim YJ Lee HM Lee HS. *In vivo* cortical spreading pattern of tau and amyloid in the Alzheimer disease spectrum. Ann Neurol. (2016) 80:247–58. 10.1002/ana.24711 27323247

[40] Maass A Landau S Baker SL Horng A Lockhart SN La Joie R. Comparison of multiple tau-PET measures as biomarkers in aging and Alzheimer's disease. Neuroimage. (2017) 157:448–63. 10.1016/j.neuroimage.2017.05.058 28587897 PMC5814575

[41] Wolters EE Golla SSV Timmers T Ossenkoppele R van der Weijden CWJ Scheltens P A novel partial volume correction method for accurate quantification of [18F] flortaucipir in the hippocampus. EJNMMI Res. (2018) 8:79 10.1186/s13550-018-0432-2 30112620 PMC6093830

[42] Mattsson N Smith R Strandberg O Palmqvist S Schöll M Insel PS. Comparing 18F-AV-1451 with CSF t-tau and p-tau for diagnosis of Alzheimer disease. Neurology. (2018) 90:e388–95. 10.1212/WNL.0000000000004887 29321235 PMC5791788

